# Meta-analysis of the efficacy and safety of OLIF and TLIF in the treatment of degenerative lumbar spondylolisthesis

**DOI:** 10.1186/s13018-024-04703-1

**Published:** 2024-04-15

**Authors:** Jing Shi, Han Wu, Fenyao Li, Jinpeng Zheng, Ping Cao, Bing Hu

**Affiliations:** Tianyou Hospital, Wuhan University of Science and Technology, Wuhan, 430064 China

**Keywords:** Lumbar interbody fusion, Degenerative lumbar spondylolisthesis, Oblique lateral lumbar interbody fusion, Transforaminal lumbar interbody fusion, Meta-analysis

## Abstract

**Objective:**

To systematically evaluate the difference in clinical efficacy between two surgical approaches, oblique lateral approach and intervertebral foraminal approach, in the treatment of degenerative lumbar spondylolisthesis.

**Methods:**

English databases, including PubMed, Cochrane, Embase, and Web of Science, were systematically searched using keywords such as "oblique lumbar interbody fusion" and "transforaminal lumbar interbody fusion." Concurrently, Chinese databases, including CNKI, WanFang data, VIP, and CBM, were also queried using corresponding Chinese terms. The search spanned from January 2014 to February 2024, focusing on published studies in both Chinese and English that compared the clinical efficacy of OLIF and TLIF. The literature screening was conducted by reviewing titles, abstracts, and full texts. Literature meeting the inclusion criteria underwent quality assessment, and relevant data were extracted. Statistical analysis and a meta-analysis of the observational data for both surgical groups were performed using Excel and RevMan 5.4 software. Findings revealed a total of 14 studies meeting the inclusion criteria, encompassing 877 patients. Of these, 414 patients were in the OLIF group, while 463 were in the TLIF group. Meta-analysis of the statistical data revealed that compared to TLIF, OLIF had a shorter average surgical duration (*P* < 0.05), reduced intraoperative bleeding (*P* < 0.05), shorter average hospital stay (*P* < 0.05), better improvement in postoperative VAS scores (*P* < 0.05), superior enhancement in postoperative ODI scores (*P* < 0.05), more effective restoration of disc height (*P* < 0.05), and better correction of lumbar lordosis (*P* < 0.05). However, there were no significant differences between OLIF and TLIF in terms of the incidence of surgical complications (*P* > 0.05) and fusion rates (*P* > 0.05).

**Conclusion:**

When treating degenerative lumbar spondylolisthesis, OLIF demonstrates significant advantages over TLIF in terms of shorter surgical duration, reduced intraoperative bleeding, shorter hospital stay, superior improvement in postoperative VAS and ODI scores, better restoration of disc height, and more effective correction of lumbar lordosis.

**Supplementary Information:**

The online version contains supplementary material available at 10.1186/s13018-024-04703-1.

## Preamble

Lumbar spondylolisthesis refers to the anterior displacement of one vertebra over another. Based on its etiology, Wiltse [1] categorized it into degenerative, traumatic, isthmic, congenital, pathological, and iatrogenic types. It has been reported that the incidence of degenerative lumbar spondylolisthesis is on the rise. While most cases of degenerative spondylolisthesis are asymptomatic, with vertebral displacement only detectable through imaging studies, a diagnosis of degenerative lumbar spondylolisthesis (DLS) is confirmed only when mechanical back pain, radicular leg pain, or neurogenic intermittent claudication is present. For patients with isolated lower back pain without neurological symptoms, conservative treatment is typically the first choice. However, for those experiencing neurogenic intermittent claudication or radiating leg pain, if standard conservative treatment fails to provide relief after more than three months, surgical intervention is generally preferred [2]. Lumbar fusion surgery stands as a commonly employed surgical treatment method [3].

Since its initial application in 1982 by Harms and Rolinger for lumbar spondylolisthesis [4], TLIF surgery has emerged as one of the most commonly utilized techniques for lumbar interbody fusion. However, with the extensive use of TLIF in managing various degenerative lumbar conditions, concerns have arisen regarding its associated severe iatrogenic injury, paraspinal muscle atrophy, as well as limitations in correcting coronal imbalance and restoring lordosis [5]. As surgical approaches continue to evolve, OLIF has gained widespread clinical application. Mayer [6] first described the anterior trans-psoas lumbar interbody fusion through the retroperitoneal space in 1997, and Silvestre [7] officially reported the OLIF technique in 2012. Since its introduction to China in 2014, OLIF has experienced rapid development [8]. OLIF avoids the need for posterior approach surgery, thus preventing damage to the posterior tension band structures and avoiding the need for bony structure resection, while also allowing for the implantation of larger fusion cages [9]. Due to its reduced invasiveness, decreased surgical blood loss, and higher fusion rates, OLIF aligns well with the evolving demands of minimally invasive surgery [10]. Currently, there is no definitive evidence or multicenter studies comparing the efficacy of the two surgical approaches in treating degenerative lumbar spondylolisthesis. Meta-analysis stands as a high-quality assessment method in evidence-based medicine. Therefore, this study aims to consolidate existing literature and conduct a meta-analysis on the therapeutic efficacy and safety of both surgical methods, with the goal of providing valuable insights for the diagnosis and treatment of degenerative lumbar spondylolisthesis in the future.

## Information and methodology

### Literature search strategy

English databases, including PubMed, Cochrane, Embase, and Web of Science, were systematically searched using keywords such as "oblique lumbar interbody fusion" and "transforaminal lumbar interbody fusion".Concurrently, Chinese databases, including CNKI, WanFang data, VIP, and CBM, were also queried using corresponding Chinese terms "斜外侧腰椎椎间融合术" and "斜外侧入路腰椎融合术".The search spanned from January 2014 to February 2024, focusing on published studies in both Chinese and English that compared the clinical efficacy of OLIF and TLIF.

### Literature inclusion and exclusion criteria

- Inclusion Criteria: (1) Studies involving adult patients (age ≥ 18 years) diagnosed with degenerative lumbar spondylolisthesis and meeting the surgical indications for OLIF or TLIF procedures. (2) Literature types comprising randomized controlled trials, retrospective studies, or prospective studies with a minimum follow-up period of three months postoperatively. (3) Literature comparing the efficacy and safety of OLIF and TLIF in treating lumbar spondylolisthesis. (4) Research outcome measures encompassing surgical duration, intraoperative blood loss, length of hospital stay, VAS score, ODI score, JOA score, disc height (DH), lumbar lordosis angle (LL), interbody fusion rate, and postoperative complication rate.
- Exclusion Criteria: (1) Studies not involving OLIF or TLIF treatment for degenerative lumbar spondylolisthesis. (2) Exclusion of systematic reviews, meta-analyses, case reports, conference reports, and other non-original research articles. (3) Studies with incomplete surgical group information or unavailable outcome measure data for extraction.

### Literature screening and data extraction

Two researchers independently conducted the literature screening process. In cases of disagreement, a third researcher was consulted to make the final decision. Information recorded included the primary author, publication year, study type, general patient characteristics, and observed outcome measures for each group.

### Quality assessment

Based on the types of literature research, two researchers independently utilized the Cochrane risk of bias tool and the Newcastle–Ottawa Scale [11] (NOS) to assess the quality of randomized controlled trials and case–control studies, respectively. In instances of disagreement in the assessment results, a third researcher was consulted for discussion and a final decision.

### Statistical treatment of literature data

Data were statistically analyzed using Excel and RevMan 5.4 software for Meta-analysis. For continuous variables such as surgical duration and blood loss, the mean difference (MD) was calculated. For categorical data like postoperative complication rates and fusion rates, the odds ratio (OR) was computed, along with the 95% confidence interval (CI). Heterogeneity was assessed using both the Chi-square (χ^2^) test and the Q test. Meta-analysis was performed using a fixed-effects model when the *P* ≥ 0.1 of heterogeneity χ2 and the Q test level I^2^ < 50% and the smaller value of I^2^ indicated less heterogeneity among the studies; when the *P* < 0.1 and the Q test I^2^ ≥ 50%, it indicated a large heterogeneity among the studies, and a random-effects model was used. The bias of each study was assessed using a funnel plot.

## Results

### Literature search screening process and results

**Fig. 1:**
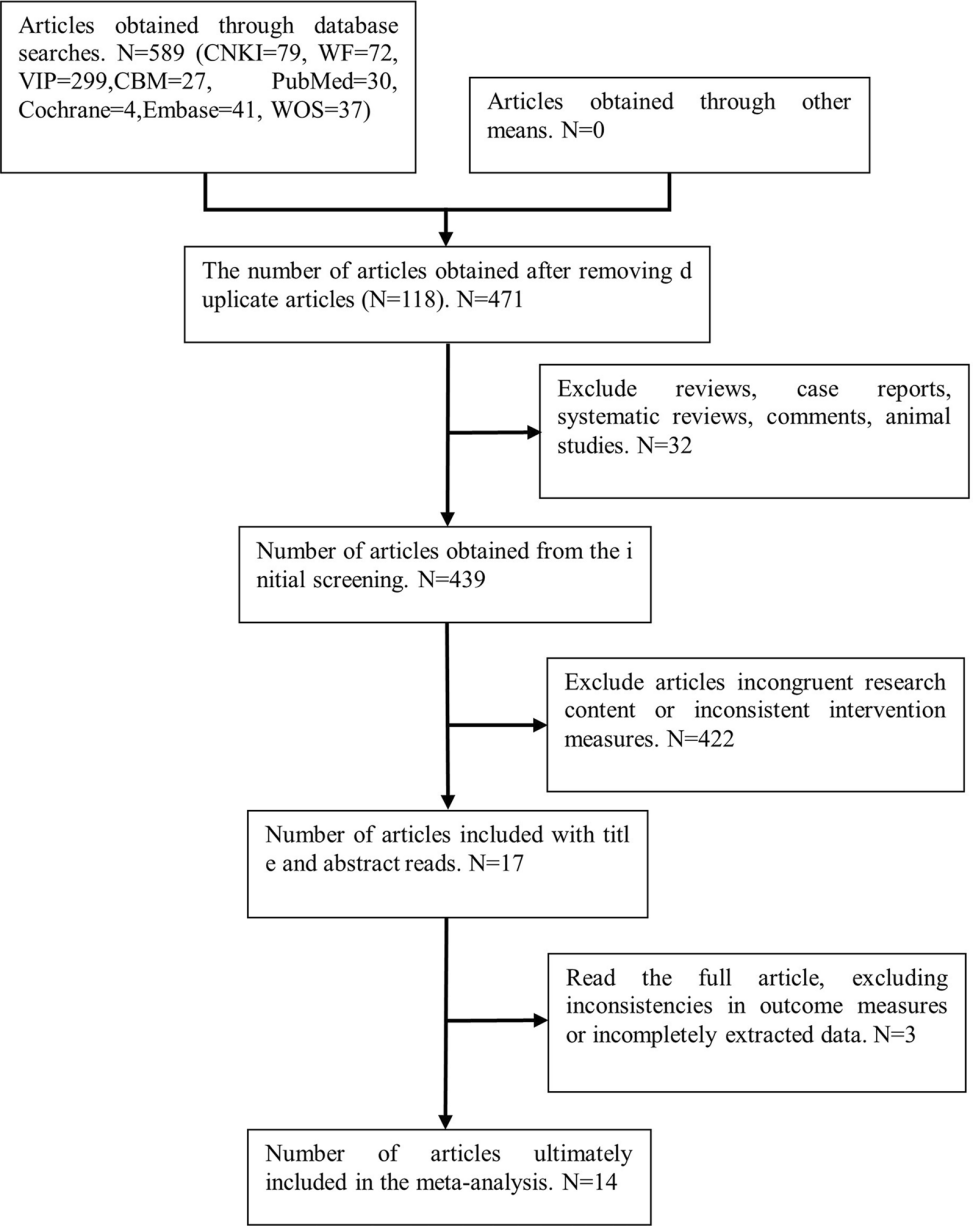
Literature screening flowchart

A total of 589 relevant articles were identified through database searches: 299 from the Weipu database, 79 from the CNKI database, 72 from the Wanfang database, 27 from the CBM database, 41 from the Embase database, 37 from the Web of Science database, 30 from the PubMed database, and 4 from the Cochrane Library database. After removing 118 duplicate articles and excluding 32 systematic reviews, meta-analyses, and case reports based on title and abstract screening, 422 articles were further excluded due to inconsistent research content or varying surgical protocols upon full-text review. Ultimately, three articles were excluded for incomplete data. A total of 14 articles met the inclusion criteria, comprising three in English and ten in Chinese. The flowchart detailing the literature selection process is presented in Fig. 1. Among the 14 included articles, one was a randomized controlled trial, while the rest were cohort studies. The cumulative sample size across these studies was 877 cases, with 414 cases in the OLIF group and 463 cases in the TLIF group. The characteristics of the included studies are summarized in Table 1.

**Table 1:** Characteristics of the included literature

First author	Year	Research design method	Gender (N)		OLIF/TLIF			Nos	Risk of biasassessment
			Males	Females	Sample size (N)	Average age (year)	Average follow-up time (months)		
Hiromitsu Takaoka	2021	Retrospective study	63	80	65/78	66 ± 12 / 71 ± 9	64.0 ± 16.2/53.0 ± 13.0	7	
Guangqing Li	2022	Retrospective study	16	56	36/36	58.52 ± 7.26 / 59.88 ± 7.04	43.13 ± 3.24/44.42 ± 4.54	7	
Renjie-Li	2021	Retrospective study	15	48	28/35	57.5 ± 10.4 / 59.3 ± 9.86	6	7	
Zhongyou Zeng	2022	Prospective study	33	82	59/56	56 ± 8 / 58 ± 6	27.0 ± 3.4	7	
Tairui Guo	2022	Retrospective study	30	24	25/29	56.5 ± 10.2	13. 9 ± 1. 9	5	
Kai Huang	2019	Retrospective study	33	23	26/30	60. 33 ± 4. 88 / 61. 04 ± 4. 84	24	7	
Hongjun Lei	2020	Randomized controlled trial	34	26	30/30	57.2 ± 4.5 / 56.8 ± 4.3	6	\	Low risk
Renjie Li	2021	Retrospective study	13	55	33/35	59.7 + 8.5 / 58.6 + 7.58	6	7	
Weibin Tie	2022	Retrospective study	14	29	20/23	59.75 ± 9.25 / 58.78 ± 8.06	22.70 ± 6.51	7	
Shengdong Wang	2021	Retrospective study	23	45	33/35	62.76 ± 6.83 / 58.51 ± 6.84	12	7	
Qiang Zhang	2019	Retrospective study	13	17	15/15	50.2 ± 7.6 / 51.5 ± 6.3	8.5 ± 2.3	7	
Shengming Wu	2019	Retrospective study	12	21	14/19	60 ± 9 / 55 ± 12	3	7	
Zhiwei Ye	2020	Retrospective study	19	26	20/25	46.14 ± 9.24 / 47.63 ± 13.28	14.3	7	
Shiqiang Qin	2023	Retrospective study	11	16	10/17	50.4 ± 7.79 / 61.7 ± 8.51	15.3 ± 3.4	7	

### Assessment of the quality of the included literature

Quality assessment of the 14 included articles [12–25] was conducted using the risk of bias assessment tool or the NOS scale. The NOS scale has a maximum score of 9, with scores above 5 indicating higher-quality literature and those above 7 considered high-quality. The assessment revealed that one randomized controlled trial was rated as low risk, with 12 articles deemed high-quality and one as higher-quality. Details of the literature quality are presented in Table 1.

### Meta-analysis results

When comparing perioperative indicators, OLIF demonstrated shorter surgical duration than TLIF (*P* < 0.05), less intraoperative blood loss (*P* < 0.05), and a shorter average hospital stay (*P* < 0.05). In terms of clinical outcomes, postoperative VAS and ODI scores were superior in the OLIF group compared to the TLIF group (*P* < 0.05). However, there was no statistically significant difference in postoperative JOA scores between the two groups (*P* > 0.05). Additionally, the OLIF group exhibited better recovery in disc height (DH) and lumbar lordosis (LL) postoperatively than the TLIF group (*P* < 0.05). Regarding surgical safety, there were no statistically significant differences in the rates of postoperative complications (*P* > 0.05) or fusion between the OLIF and TLIF groups (*P* > 0.05). In summary, compared to TLIF surgery, OLIF demonstrates superior clinical efficacy in treating degenerative lumbar spondylolisthesis, as shown in Table 2 (The analysis process, meta-analysis forest plot, and funnel plot can be found in Additional file 1).

**Table 2:** Incorporating the meta-analysis results of the include research literature

Outcome measures	Number of OLIF	Number of TLIF	Heterogeneity test (I^2^)%	Test model	Combined statistics	Values of combined statistics (95% CI)	P
Surgical duration [12, 13, 15, 16, 18–25]	313	349	97	Random-effects model	MD	−21.58 [−31.86, −11.30]	< 0.05
Intraoperative blood loss [12, 13, 15, 16, 18–25]	313	349	100	Random-effects model	MD	−117.09 [−181.46, −52.72]	< 0.05
Length of hospital stay [14–16, 18–20, 23, 25]	200	230	93	Random-effects model	MD	−3.90 [−2.60, −2.12]	< 0.05
Postoperative VAS score [12–25]	355	407	18	Fixed-effects model	MD	−0.13 [−0.21, −0.06]	< 0.05
Postoperative ODI score [12–16, 18–25]	334	370	32	Fixed-effects model	MD	−0.58 [−0.95, −0.21]	< 0.05
Postoperative JOA score [13, 22, 23]	62	77	71	Random-effects model	MD	0.57 [0.36, 1.50]	> 0.05
Postoperative DH [12, 13, 16–24]	348	386	91	Random-effects model	MD	1.19 [0.55, 1.84]	< 0.05
Postoperative LL [16, 17, 19–25]	200	220	27	Fixed-effects model	MD	1.82 [0.88, 2.75]	< 0.05
Postoperative complication rate [12, 13, 15–25]	384	433	23	Fixed-effects model	OR	1.05 [0.72, 1.52]	> 0.05
Interbody fusion rate [12, 13, 16, 22–24]	108	113	0	Fixed-effects model	OR	1.84 [0.57, 5.97]	> 0.05

## Discussion

### Background on the treatment of degenerative lumbar spondylolisthesis

The majority of patients with DLS can be effectively managed through non-surgical treatments. These primarily include bed rest, medication, and physical therapy. Bed rest helps alleviate intervertebral joint stress, providing relief from lower back pain and radicular symptoms. During acute episodes of lower back pain, non-steroidal anti-inflammatory drugs (NSAIDs) or local block therapy can be used for rapid symptom relief. Physical therapies, such as localized heating and the use of lumbar braces, aim to relax the surrounding spinal muscles to alleviate pain.

Non-surgical treatments are preferred in clinical settings due to their convenience and relative safety. They are generally well-received, and most patients with simple lower back pain experience significant relief. However, for those with more severe symptoms, the relief may be short-lived. If non-surgical treatments prove ineffective after three months, or if the symptoms significantly impact daily work and life, clinicians often lean towards recommending surgical intervention [26].

Transforaminal lumbar interbody fusion (TLIF) has become one of the most commonly performed procedures for lumbar interbody fusion due to its proven efficacy. It is widely utilized in clinical practice, enabling joint facet resection, spinal canal decompression, and vertebral fusion through a unilateral intervertebral approach. The procedure aims to restore intervertebral height, alleviate nerve compression, and reconstruct lumbar stability. Importantly, TLIF preserves the anterior longitudinal ligament and contralateral posterior longitudinal ligaments as well as the contralateral vertebral plate. This approach minimizes traction on the traversing nerve roots and dural sac, resulting in fewer postoperative neurological complications. However, the traditional posterior midline approach used in TLIF necessitates the detachment and retraction of the multifidus muscles bilaterally, leading to muscle damage, postoperative scar formation, and denervation of paraspinal muscles. These effects can directly weaken the spinal flexion force, resulting in postoperative lower back pain and potential complications like failed back surgery syndrome [27]. Furthermore, compromising the integrity of the spinal posterior column may contribute to adjacent segment degeneration (ASD) in postoperative patients [28].

The OLIF procedure utilizes a working channel placed in the retroperitoneal space, anterior to the psoas muscle, eliminating the need to dissect the psoas muscle as in TLIF, thereby reducing the risk of bleeding and injury. This approach offers a larger operating space, enabling anterior clearance of the intervertebral disc without nerve traction. Additionally, it allows for the implantation of larger interbody fusion devices, potentially improving fusion rates [29]. Some studies suggest that compared to traditional posterior lumbar interbody fusion (PLIF) surgeries, OLIF achieves superior decompression results with reduced trauma and faster postoperative recovery [30]. This study will discuss the clinical efficacy and safety of OLIF and TLIF in treating degenerative lumbar spondylolisthesis, focusing on perioperative indicators, surgical outcomes, and the incidence of complications.

### Analysis of findings

#### Analysis of perioperative data for both surgical modalities

A total of 12 studies were included to compare the differences in surgical duration between the two groups. All studies utilized pedicle screw fixation, with three of them combining OLIF surgery with anterior or lateral vertebral body screw fixation, while the others employed posterior pedicle screw fixation. Some research suggests that combining internal fixation during decompression and fusion can enhance efficacy and fusion rates. However, there is no consensus on whether OLIF surgery should use lateral or posterior internal fixation. Meta-analysis results indicated that compared to TLIF, OLIF surgery had a shorter duration, with statistically significant differences (*P* < 0.05).

Twelve studies compared the differences in intraoperative blood loss between the two groups. Meta-analysis results showed that the OLIF group had significantly less intraoperative blood loss (*P* < 0.05). Surgical bleeding is associated with the size of the surgical incision and the choice of surgical approach, while the proficiency of the surgeon can also influence both the surgical duration and blood loss. The OLIF surgery, entering between the retroperitoneum and the psoas muscle, results in minimal damage to the surrounding soft tissues of the spine. It allows for addressing intervertebral disc tissue from the anterior aspect of the spine, thus avoiding disruption to the posterior bony structures of the spine [31]. However, during OLIF surgery, there is a risk of damaging the blood vessels in front of the vertebra, leading to potentially fatal massive bleeding. Therefore, avoiding vascular injury is crucial for reducing intraoperative blood loss.

Eight studies were included to compare the hospitalization durations between the two groups. Meta-analysis results indicated that the OLIF group had a shorter average hospital stay (*P* < 0.05). Compared to OLIF, TLIF involves longer incisions, potentially damaging the paraspinal muscles, and necessitates the removal of facet joints, compromising the stability of the posterior vertebral column. Consequently, the recovery period is extended, leading to a longer hospital stay in the TLIF group. In summary, in terms of reducing intraoperative blood loss, shortening surgical and hospitalization times, the OLIF group outperforms the TLIF group. However, the heterogeneity tests for the aforementioned three aspects indicated significant differences, the research team's analysis suggests that the above variations may be related to the varying proficiency levels of the surgeons.

#### Comparative analysis of the clinical efficacy of the two surgical approaches

Fourteen studies were included in this analysis comparing postoperative VAS scores between the two groups. The OLIF group demonstrated a more pronounced improvement in postoperative VAS scores (*P* < 0.05). This could be attributed to the smaller incision and shorter surgical duration associated with OLIF, which also avoids excessive traction on the paraspinal muscle tissues, leading to faster postoperative recovery and reduced pain. Thirteen studies were analyzed to compare postoperative ODI scores between the two groups. The meta-analysis results consistently indicated that the OLIF group exhibited superior improvements in pain relief and lumbar function recovery compared to the TLIF surgery group. Among all included studies, there was no statistically significant difference in preoperative pain scores between the two groups (*P* > 0.05). However, both groups showed significant improvements in postoperative follow-up VAS and ODI scores compared to preoperative scores (*P* < 0.05), suggesting that both procedures effectively alleviated pain symptoms in DLS patients. Nonetheless, given the subjective nature of pain scores, further large-scale clinical data are needed in the future to reduce potential subjective biases.

This study analyzed 11 articles comparing the recovery of disc height (DH) postoperatively. Both groups of patients in the included studies exhibited varying degrees of preoperative DH loss. After surgery, the final follow-up in each study indicated a significant improvement in DH compared to preoperative levels (*P* < 0.05). This suggests that both surgical techniques effectively restored DH through Cage implantation. Notably, the Cage used in OLIF was larger, resulting in better postoperative intervertebral space recovery than that in the TLIF group (*P* < 0.05). For postoperative lumbar lordosis angle (LL), data from nine studies were included. Both groups initially presented with a loss of physiological lumbar curvature. Follow-up assessments in the literature consistently showed postoperative LL correction in both groups (*P* < 0.05), indicating the effectiveness of both techniques in restoring physiological lumbar curvature. Furthermore, the OLIF group demonstrated superior LL correction compared to the TLIF group (*P* < 0.05). OLIF surgery allows for the implantation of a larger Cage, facilitating a better expansion of the intervertebral space and achieving indirect decompression of the nerve roots. The key to correcting the lumbar lordosis angle lies in restoring the height of the intervertebral space [32]. However, it is crucial to note that if there is an excessive emphasis on restoring the normal intervertebral space height and an oversized interbody fusion device is chosen, patients may be at risk of postoperative complications such as endplate injury and cage subsidence after early rehabilitation activities [33].

Postoperative fusion rates were compared across 6 studies, with 2 studies reporting a 100% fusion rate for both procedures. The remaining 4 studies, analyzed through meta-analysis, found no significant difference in fusion rates between the two procedures (*P* = 0.31 > 0.05). OLIF allows the use of larger interbody fusion devices, theoretically leading to higher fusion rates compared to TLIF surgery. However, the interbody fusion rate after lumbar fusion surgery is influenced by factors such as patient nutritional status, the size of the interbody fusion device, and the fused segments [34]. This meta-analysis, limited by a small number of included studies, cannot verify this conclusion.

In summary, both OLIF and TLIF surgeries demonstrate favorable clinical efficacy in terms of fusion rates. However, OLIF exhibits superior clinical outcomes compared to TLIF in terms of improvement in VAS and ODI scores, restoration of disc height (DH), and correction of lumbar lordosis (LL).

#### Comparative analysis of safety/complication rates between the two surgical approaches

This study encompassed 13 literature sources comparing the postoperative complication rates between the two surgical methods. According to the Meta-analysis, there was no significant difference in the incidence of surgical complications between the two techniques (*P* = 0.81 > 0.05). The overall complication rate in the OLIF group was 16.67%. This primarily consisted of transient muscle weakness and thigh numbness at 8.33%, and endplate injury at 4.17%. In contrast, the TLIF group exhibited a total complication rate of 16.17%. This was mainly characterized by transient thigh numbness/pain at 4.16%, cerebrospinal fluid leakage at 3.93%, and poor postoperative wound healing at 2.77%.Although the OLIF approach, utilizing a lateral approach between the psoas muscle and the peritoneum, reduces the risk of nerve root damage and cerebrospinal fluid leakage [35], it does present a higher rate of endplate injuries compared to TLIF. The occurrence rate of surgical complications serves as an indicator of surgical safety, where skilled surgical procedures can prevent major vascular injuries and permanent damages. Upon comparative analysis, there was no significant difference in the safety profiles between the two surgical techniques. With the ongoing advancements in robotic-assisted surgeries, it is anticipated that the safety of these procedures will further improve [35].

### Limitations of this study

Limitations of this study include: (1) A total of 14 literature studies were included, with a limited number of randomized controlled trials and small sample sizes in each study, potentially affecting the reliability of the research. Future studies should incorporate larger sample sizes and more randomized controlled trials; (2) Variations in surgical expertise among different surgeons might result in significant heterogeneity in the literature findings; (3) Inconsistencies in the postoperative follow-up durations across the included studies currently prevent a comparison of the long-term effectiveness and safety between OLIF and TLIF.

### Supplementary Information

**Additional  file 1: **Results of the meta-analysis of the efficacy and safety of OLIF and TLIF in the treatment of degenerative lumbar spondylolisthesis.

## Data Availability

The datasets used and/or analyzed during the current study are available from the corresponding author upon reasonable request.
