# Supplementary material for: Meta-analysis of the efficacy and safety of OLIF and TLIF in the treatment of degenerative lumbar spondylolisthesis
Source: J Orthop Surg Res. 2024 Apr 15;19:242. doi: 10.1186/s13018-024-04703-1 (PMC11020183; doi:10.1186/s13018-024-04703-1)
Supplement: Supplementary file 1 — Additional file 1: Results of the meta-analysis of the efficacy and safety of OLIF and TLIF in the treatment of degenerative lumbar spondylolisthesis. [file 13018_2024_4703_MOESM1_ESM.docx]

**Meta-analysis results**

**1.** **Surgical duration comparison**

There are 12 articles included in the literature(1-12) comparing the surgical times of two groups. After conducting a heterogeneity test (I^2^=97%>50%, P<0.1), it was observed that there was significant heterogeneity among the studies. Through sensitivity analysis, it was found that the removal of any single study did not significantly impact the overall heterogeneity. Proficiency in surgical techniques was identified as a major factor influencing surgical time, thus contributing to the observed heterogeneity. To address this, a random-effects model was chosen for the Meta-analysis. The OLIF group exhibited significantly shorter surgical times, with statistical significance (Z=4.12, MD=-21.58, 95% CI: -31.86~-11.30, P<0.05) (Figure 1).


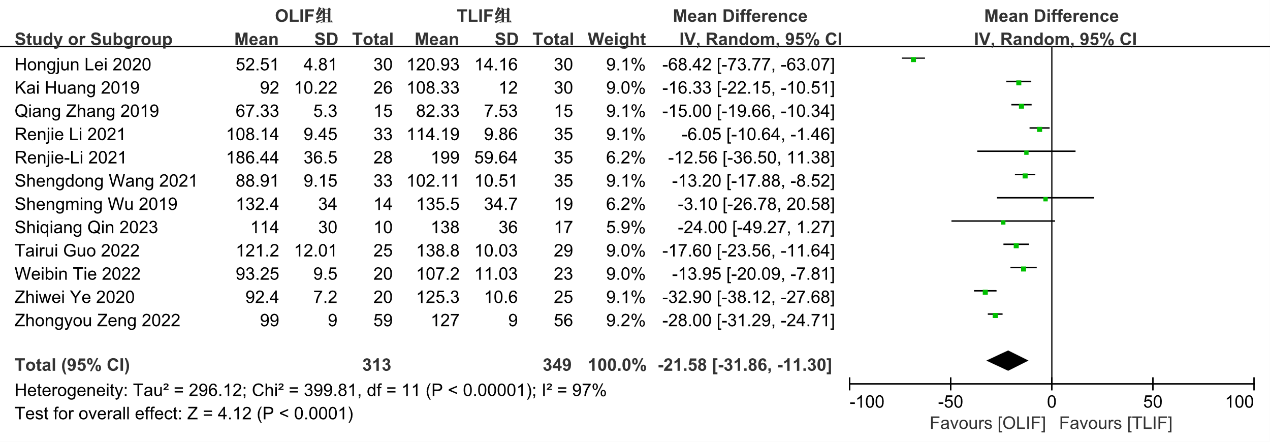


Fig. 1 Forest plot comparing Surgical duration

**2. Intraoperative blood loss comparison**

There are 12 articles included in the literature(1-12) comparing the intraoperative blood loss between two groups. Following a heterogeneity test (I^2^=100%>50%, P<0.1), substantial heterogeneity among the studies was observed. Consequently, a random-effects model was selected for the Meta-analysis. The results indicated that the OLIF group experienced significantly less surgical bleeding, with statistical significance(Z=3.57, MD=-117.09, 95%CI:-181.46~-52.72,P<0.05)(Figure2).

**
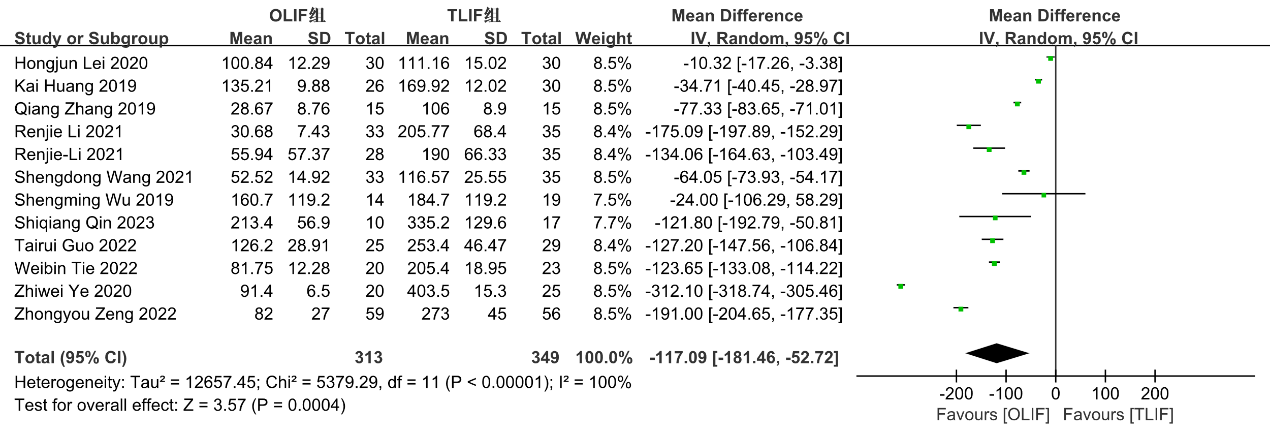
**

Fig. 2 Forest plot comparing Intraoperative blood loss

**3. Hospitalization duration comparison**

Eight articles(1, 2, 4-6, 8, 10, 11) were reviewed for the average length of hospital stay. Following a heterogeneity test, where I^2^=93%>50% and the Q-test yielded P<0.1, substantial heterogeneity among the studies was identified. To address this variability, a random-effects model was employed for the Meta-analysis. The findings revealed that the OLIF group exhibited significantly shorter average hospital stays compared to the TLIF group, with statistical significance (Z=6.62, MD=-3.01, 95%CI:-3.90~-2.12, P<0.05)(Figure 3).


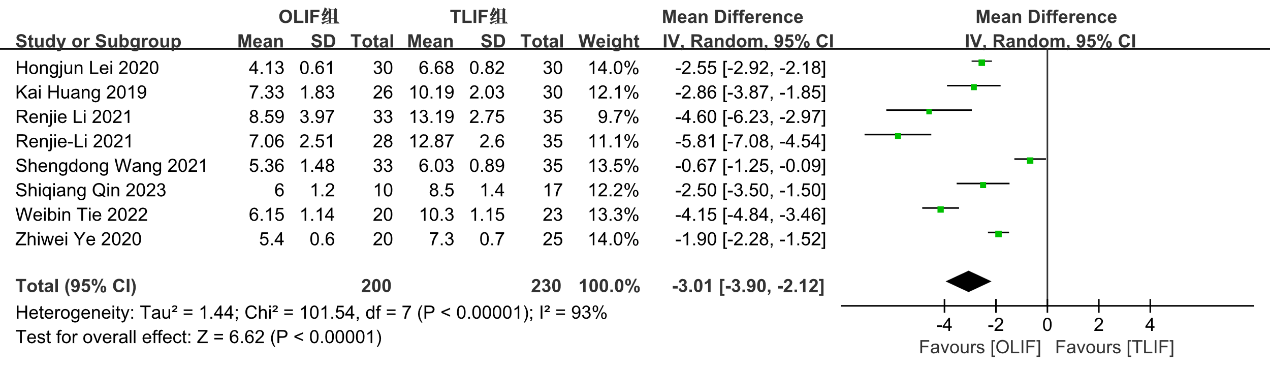


Fig. 3 Forest plot comparing Hospitalization duration

**4. Postoperative VAS score comparison**

Fourteen articles(1-14) were reviewed for the comparison of postoperative VAS scores. Following a heterogeneity test, where I^2^=71%>50% and the Q-test yielded P<0.1, significant heterogeneity among the studies was identified. Sensitivity analysis was conducted, and upon removing the study by Zongyou Zeng in 2022, a reevaluation of heterogeneity showed that the remaining 13 studies exhibited no significant heterogeneity (P=0.27>0.1, I^2^=18%). Consequently, a fixed-effects model was employed to merge the effect sizes. In the treatment of degenerative lumbar spondylolisthesis, the postoperative VAS improvement in the OLIF group surpassed that in the TLIF group, and the difference between the two groups was statistically significant (Z=3.48,MD=-0.13,95%CI:-0.21~-0.06,P<0.05)(Figure 4).


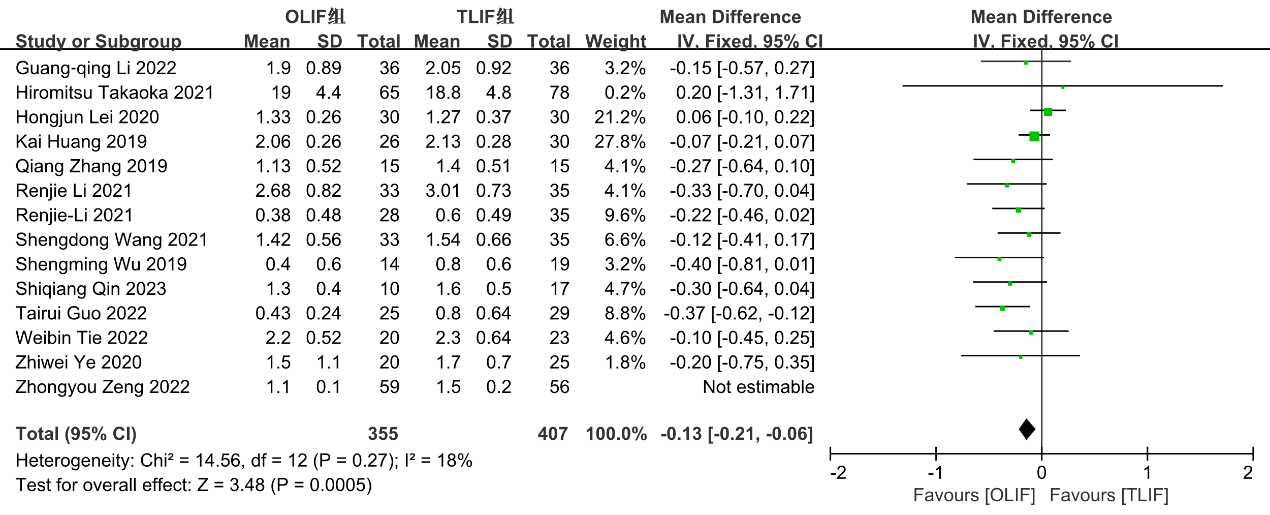


Fig. 4 Forest plot comparing Postoperative VAS scores

**5. Postoperative ODI score comparison**

Thirteen articles(1-13) were reviewed for the comparison of postoperative ODI scores. Following a heterogeneity test, where I^2^=61%>50% and the Q-test yielded a significant result (P=0.002<0.1), considerable heterogeneity among the studies was identified. Sensitivity analysis was conducted, revealing that the removal of the study by Qiang Zhang in 2019 resolved the heterogeneity issue. Subsequent heterogeneity testing for the remaining 11 studies showed no significant heterogeneity (P=0.14>0.1, I^2^=32%). Therefore, a fixed-effects model was employed. In the treatment of degenerative lumbar spondylolisthesis, the postoperative ODI score improvement in the OLIF group surpassed that in the TLIF group, and the difference between the two groups was statistically significant (Z=3.10, MD=-0.58, 95%CI:-0.95~-0.21, P=0.002<0.05)(Figure5).

**
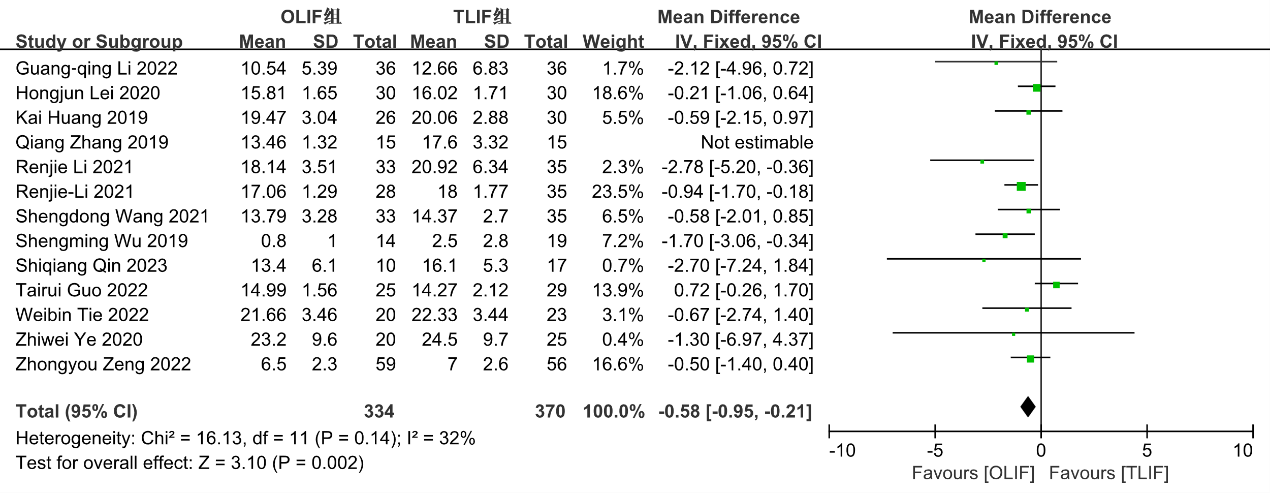
**

Fig. 5 Forest plot comparing postoperative ODI scores

**6. Postoperative JOA score comparison**

Three articles(6, 9, 10) reported postoperative JOA scores. Following a heterogeneity test, where I^2^=71%>50% and the Q-test yielded a significant result (P=0.03<0.1), substantial heterogeneity among the studies was identified. To address this variability, a random-effects model was employed for the Meta-analysis. In the treatment of degenerative lumbar spondylolisthesis, there was no statistically significant difference in postoperative JOA score improvement between the OLIF and TLIF groups (Z=1.20, MD=0.57, 95% CI:-0.36~1.50, P=0.23>0.05)(Figure6).

**
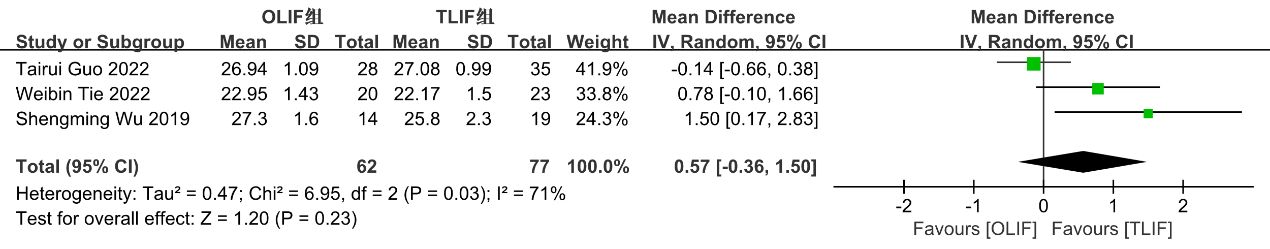
**

Fig. 6 Forest plot comparing Postoperative JOA scores

**7. Postoperative DH comparison**

Eleven articles(3-7, 9-14) were reviewed to compare postoperative disc height (DH) between two groups. Following a heterogeneity test, where I^2^=91%>50%, and P<0.1, substantial heterogeneity among the studies was identified. Analysis suggested that the variability might be attributed to differences in examination methods across studies, as DH measured from standing X-ray images tended to be slightly lower than those obtained from supine position imaging methods like CT. Sensitivity analysis showed that the removal of any single study had minimal impact on overall heterogeneity. Therefore, a random-effects model was employed for the Meta-analysis. The results demonstrated that the OLIF group exhibited superior recovery of DH compared to the TLIF group, and the difference was statistically significant(Z=3.64, MD=1.19, 95%CI:0.55~1.84, P<0.05)(Figure7).

**
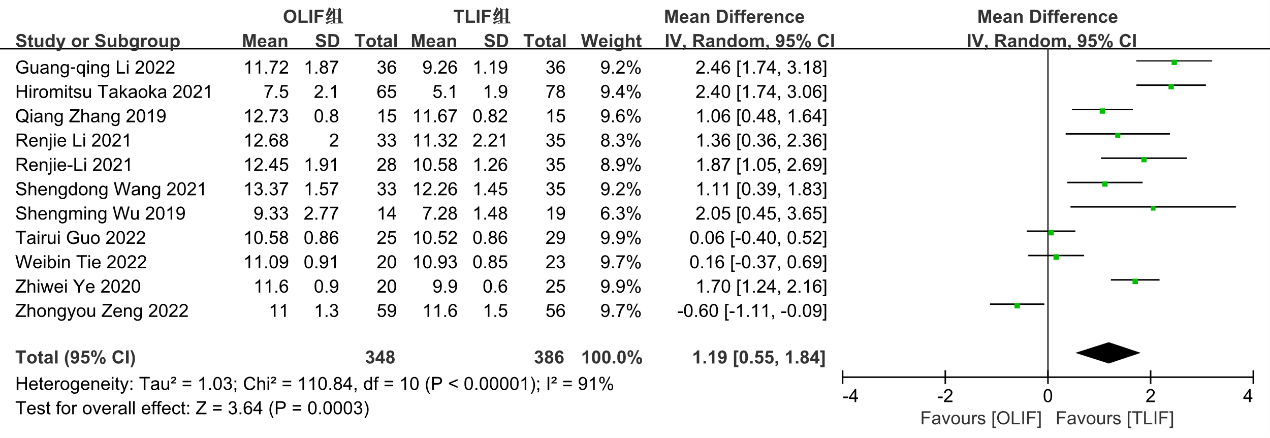
**

Fig. 7 Forest plot comparing Postoperative DH

**8. Postoperative LL comparison**

Nine articles(4, 6, 8-14) were reviewed regarding postoperative lumbar lordosis (LL). Following a heterogeneity test, where I^2^=27%<50% and the Q-test yielded a non-significant result (P=0.22>0.1), indicating low heterogeneity, a fixed-effects model was employed for the Meta-analysis. The results revealed that the OLIF group exhibited a significantly better improvement in postoperative LL compared to the comparison group, and the difference was statistically significant (Z=3.82, MD=1.82, 95% CI:0.88~2.75, P<0.05)(Figure8).

**
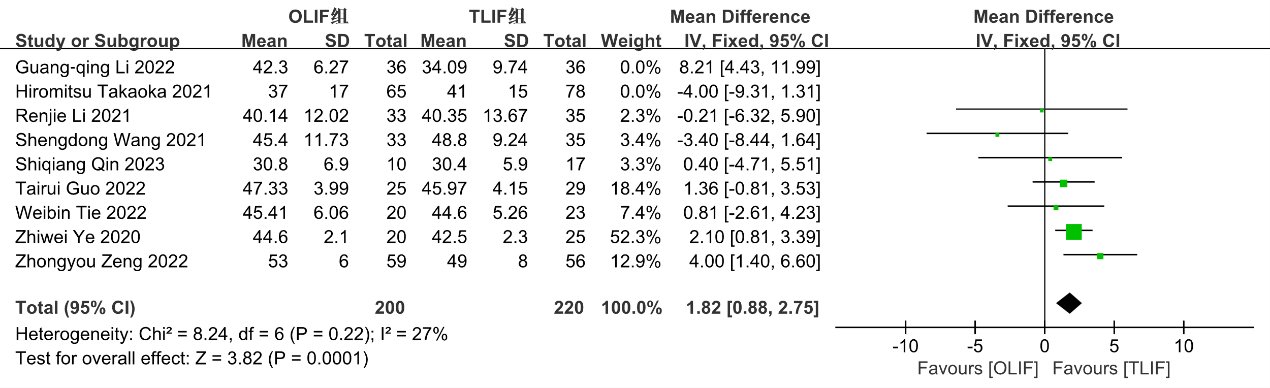
**

Fig. 8 Forest plot comparing Postoperative LL

**9. Postoperative complications comparison**

Thirteen articles(2-14) were reviewed regarding postoperative complications. Following a heterogeneity test, where I^2^=23%<50% and the Q-test yielded a non-significant result (P=0.21>0.1), indicating low heterogeneity, a fixed-effects model was employed for the Meta-analysis. The results revealed no statistically significant difference in postoperative complications between the two groups in the treatment of degenerative lumbar spondylolisthesis (Z=0.24, OR=1.05, 95%CI:0.72~1.52, P=0.81>0.05)(Figure9).


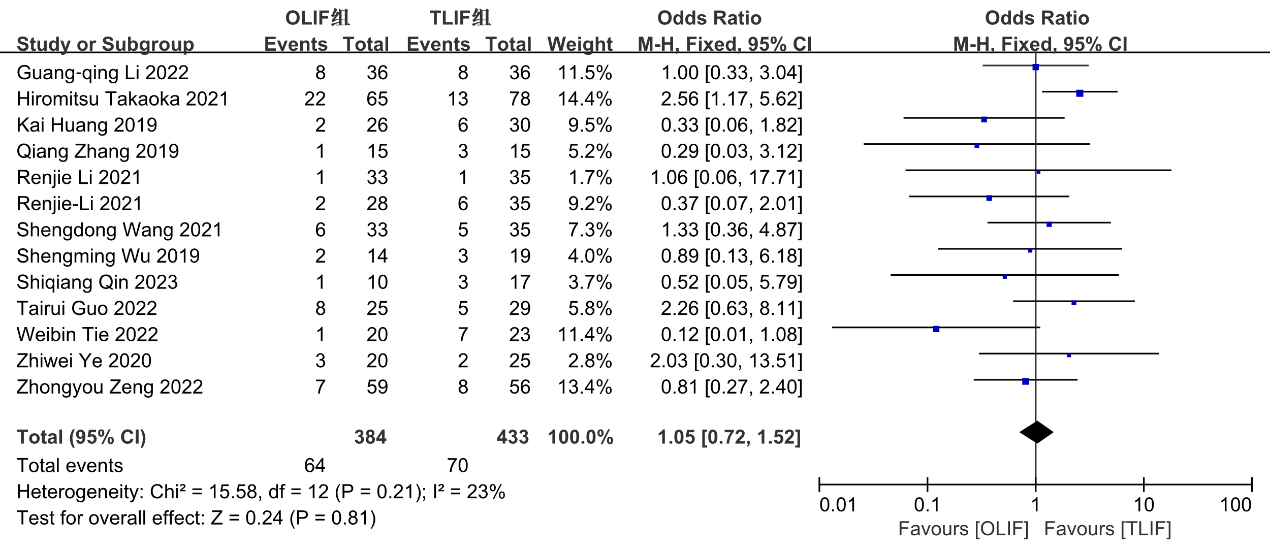


Fig. 9 Forest plot comparing Postoperative complications

**10. Postoperative fusion rate comparison**

Six articles(3, 7, 9-12) were reviewed regarding postoperative fusion rates. Notably, the fusion rates reported by Ye Zhiwei in 2020 and Guo Tairui in 2022 were both 100%. For the remaining four studies, a heterogeneity test was conducted (I^2^=0%<50%, P=0.58>0.1), indicating low heterogeneity. Consequently, a fixed-effects model was employed for the Meta-analysis. The results showed no statistically significant difference in postoperative fusion rates between the two groups (Z=1.02, OR=1.84, 95%CI:0.57~5.97, P=0.31>0.05)(Figure10).


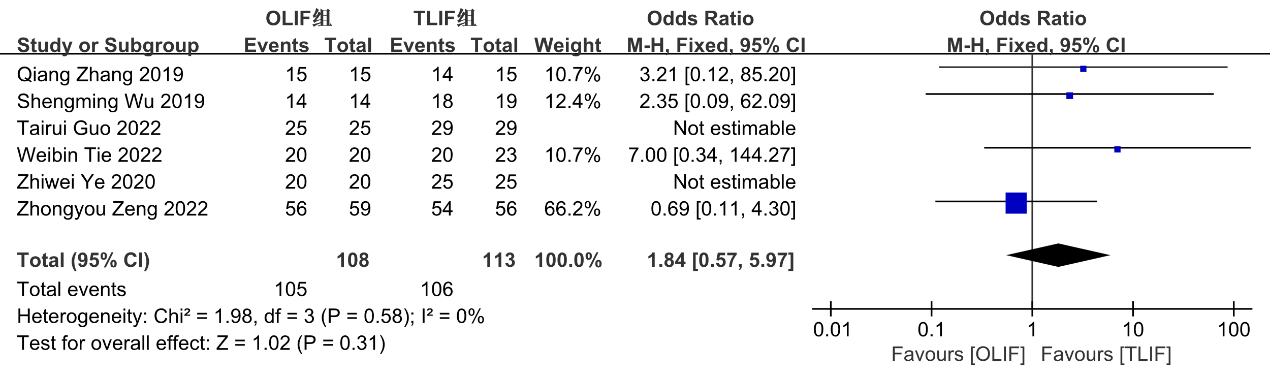


Fig. 10 Forest plot comparing Postoperative fusion rate

**Literature bias**

A funnel plot was generated using RevMan 5.4 software to assess the potential publication bias in the results of the included studies. The scatter plots for the data from the 14 articles are presented in the following figure. It is evident that the distribution of data in each funnel plot is uneven, indicating a substantial presence of publication bias across all studies (Figure 11).

a
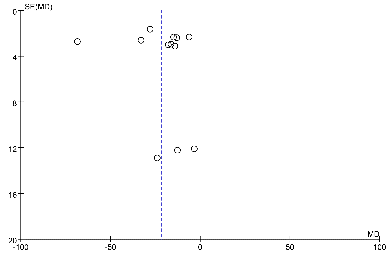
b
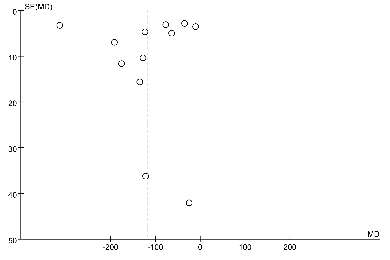
c**
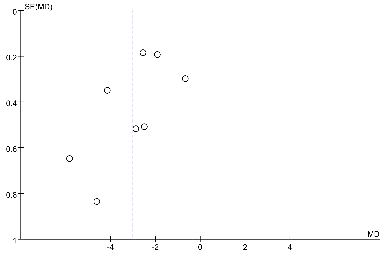
**

d
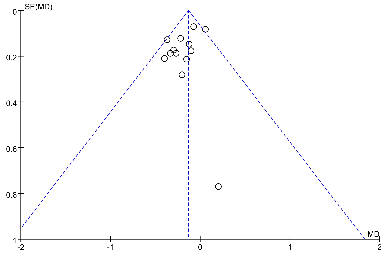
e
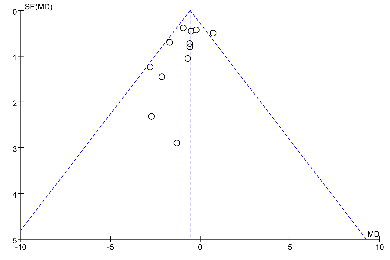
f
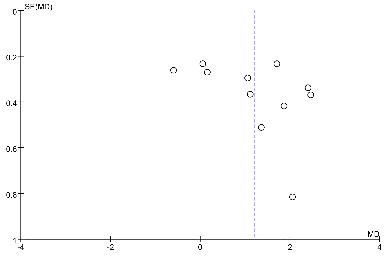


g
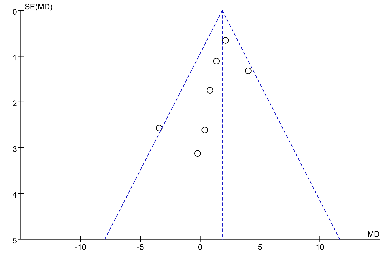
h
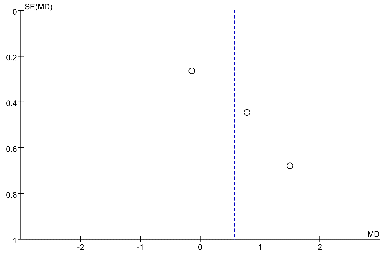
i
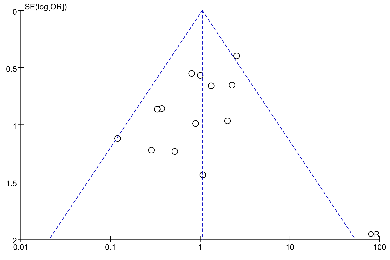


j
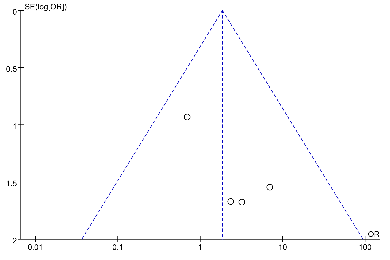


Fig. 11 Publication bias funnel plot (a: Surgical duration; b: Intraoperative blood loss; c: Hospitalization duration; d: Postoperative VAS scores; e: Postoperative ODI scores; f: Postoperative DH; g: Postoperative LL; h: Postoperative JOA scores; i: Postoperative complications; and j: Postoperative fusion rate)

Bibliography

1. Hongjun L, Zhongxian C, Zhaozong F, Shanshan X. Comparison of recent results between OLlF and TLlF in the treatment of lumbar spondylolisthesis. Chinese and Foreign Medical Research. Chinese and Foreign Medical Research. 2020;18(34):61-3.

2. Kai H, Jinhua Y, Wenming P, Wei Z, Yixin S. Comparison of the efficacy of oblique lateral approach and transforaminal lumbar interbody fusion in degenerative lumbar spondylolisthesis. The Journal of Cervicodynia And Lumbodynia. 2020;41(02):237-9.

3. Qiang Z, Guodong S, HongWei D, Ning X, HongYang S, XiaoHai S. Recent outcomes of oblique lateral/transforaminal approach lumbar interbody fusion for degenerative lumbar spondylolisthesis. Journal of Spine Surgery. 2019;17(06):369-73+93.

4. Renjie L. Preliminary efficacy analysis of OLIF and TLIF in the treatment of degenerative lumbar spondylolisthesis [M.S.]: Suzhou University; 2021.

5. Renjie L, Xiaofeng S, Xuefeng L, Yijie L, Weimin J. Comparison of clinical outcomes and spino-pelvic sagittal balance in degenerative lumbar spondylolisthesis: Minimally invasive oblique lumbar interbody fusion (OLIF) versus transforaminal lumbar interbody fusion (TLIF). Medicine. 2021;100(3).

6. Shengdong W, Peng C, Shaowen D, Xiang L, Kaishan Y. Comparison of recent efficacy of oblique lateral approach and transforaminal approach for interbody fusion in the treatment of single-segment lumbar spondylolisthesis. Journal of Xi'an Jiaotong University(Medical Sciences). 2021;42(6).

7. Shengming W. Comparison of short-term efficacy between oblique lateral approach interbody fusion and transforaminal interbody fusion for the treatment of single-segment lumbar degenerative disease [M.S.]: Fujian Medical University; 2019.

8. Shiqiang Q, Yuqing S, Zhao L, Wenzuo N. Treatment of lumbar 5 vertebral body slippage using interbody fusion via oblique lateral approach combined with posterior internal fixation. Chinese Journal for Clinicians. 2023;51(09):1077-80.

9. TaiRui G, Hui S, Rui C, Tianyu S, Zhichen X. Comparison of the efficacy of posterior interbody fusion and oblique lateral lumbar fusion combined with posterior pedicle screw internal fixation for degenerative lumbar spondylolisthesis. Medical Journal of Wuhan University. 2022;43(01):133-8.

10. Weibin T, Hui N, Hongjian L. Oblique lateral lumbar interbody fusion with pedicle fixation in high-grade degenerative lumbar spondylolisthesis. Orthopedic Journal of China. 2022;30(13).

11. Zhiwei Y, Shijie Z, LongHai P, Rongping Z. Comparison of the efficacy of OLIF and TLIF in the treatment of L4-5 single-segment degenerative lumbar spondylolisthesis. Practical Clinical Medicine. 2020;21(01):19-24.

12. Zhongyou Z, Jianqiao Z, Yongxing S, Wei Y, Shunwu F, Xiangqian, et al. A comparative study of oblique lateral approach and posterior fusion for the treatment of lumbar spondylolisthesis of I-II degree. Chinese Journal of Geriatric Orthopaedics and Rehabilitation (Electronic Edition). 2022;8(2).

13. Guangqing L, Tong T, Linfeng W. Comparative analysis of the effects of OLIF and TLIF on adjacent segments after treatment of L4 degenerative lumbar spondylolisthesis. Journal of Orthopaedic Surgery and Research. 2022;17(1).

14. Hiromitsu T, Kazuhide I, Yawara E, Yasuhiro S, Takeo F, Satoshi M, et al. Comparison between intervertebral oblique lumbar interbody fusion and transforaminal lumbar interbody fusion: a multicenter study. Scientific Reports. 2021;11(1).
